# Characteristics and factors influencing the expansion of urban construction land in China

**DOI:** 10.1038/s41598-024-67015-8

**Published:** 2024-07-11

**Authors:** Xiaodong Zhang, Haoying Han

**Affiliations:** 1 College of Water Resources and Architectural Engineering, Tarim University, Alaer, 843300 China; 2 College of Civil Engineering and Architecture, Zhejiang University, Hangzhou, 310058 China; 3 Faculty of Innovation and Design, City University of Macau, Macau, China

**Keywords:** **E**xpansion of construction land, Land use, Urban sprawl, Influencing factors, Spatial distribution characteristics, Sustainable development, China, Urban ecology, Sustainability

## Abstract

As a new product of rapid urbanization, the sprawl of urban construction land can objectively reflect urban land use efficiency, which is of great significance to China's new urban construction. This study aimed to summarize the expansion patterns and utilization efficiency of urban construction land in China from the perspectives of the status, speed and trends of expansion, and to uncover the key factors that lead to the differential distribution of the expansion of construction land. It can also provide land management experience for other countries with rapid expansion of construction land. The results show the following. (1) The expansion of China's construction land presents a "point–line–plane" pattern of evolution, forming changing stages of point-like aggregation, linear series and planar spread. (2) China's construction land shows the characteristics of disorderly spread, a low utilization rate and low output efficiency. The speed of expansion presents clear characteristics of being high in the east and low in the west, mostly concentrated in the Yangtze River Delta, Pearl River Delta and the Beijing–Tianjin–Hebei urban agglomeration. Shanghai, Beijing, Shenzhen and Guangzhou have the highest intensity of construction land use. In Shandong Peninsula and eastern coastal areas, the intensity of the construction land use is generally high. In Xinjiang and Xizang, the intensity of construction land use is relatively low. (3) The urban economic level, population size, industrial structure, foreign investment and land policies have significant effects on the spatial distribution of the expansion of construction land.

## Introduction

With the acceleration of globalization and development, the rapid expansion of urban construction land has become an inevitable trend. As a country with one of the fastest rates of urbanization in the world, China's urbanization rate has increased from 26.44% in 1990 to 63.9% in 2020^1,2^. Urbanization has entered the mature stage rapidly. Driven by urbanization and industrialization, the scale of urban construction land has also shown a rapid growth trend^3^. By calculating the data of this study, we found that the urban construction land increased from 10,264 km^2^ to 58,355.3 km^2^ from 1990 to 2020, with an average annual growth of 1603.04 km^2^. The rapid expansion of urban construction land has become the main feature of land use change in China at present and has been so for a long time^4^. It is estimated that the urbanization rate of China will reach 73.9% in 2035^5^. According to the current expansion rate, China will need to increase its construction land area by about 1500 km^2^ every year, and the supply of urban construction land area will face huge challenges^6^. At the same time, the rapid expansion of construction land leads to a series of problems such as the loss of farmland, deterioration of the ecological environment and traffic congestion, which affect the sustainable regional development^7^. How to control and restrict the rapid expansion of China's land from the root has become an urgent problem to be solved^8,9^. In light of this, exploring the characteristics and factors influencing the rapid expansion of urban construction land in China has gradually become a hot field of research.

In recent years, scholars have carried out a considerable amount of research on the expansion of urban construction land and the effects and driving forces that control expansion^10,11^. Firstly, regarding the expansion of urban construction land and its impact, scholars have found that the impact of such expansion has a two-way effect, with both negative and positive effects^12,13^. Under the rapid promotion of industrialization, urban construction land has spread to the suburbs on a large scale^14,15^. Urban sprawl creates many social and environmental problems, such as traffic congestion, environmental pollution, loss of cultivated land, fragmented land use, housing shortages and deterioration of the ecological environment^16,17^. In order to solve the problem of the disorderly sprawl of urban construction land, some scholars have begun to study the driving mechanisms of the expansion of urban construction land^18,19^. Most studies have shown that the expansion of urban construction land is affected by the economy, population, policies and natural conditions^20,21^. The driving force of different factors in different types of urban expansion is obviously different. Among them, population growth and economic development are the main factors influencing the expansion of urban construction land^22,23^. The policy system also plays an important role in the expansion of urban land, especially in China^24^. Major national development strategies, regional development policies and local development of construction zones will promote rapid urban expansion^25^. Natural factors are also important factors affecting the rapid expansion of urban construction land^26^. It is found that superior natural conditions and geographical locations near roads and coastlines contribute to the rapid expansion of urban construction land^27^. In light of these driving factors, scholars have carried out research on strategies for controlling the expansion of urban land^28,29^. The development of the concepts of "compact cities" and "smart growth" have been put forward to guide the rational expansion of urban construction land and control urban sprawl^30,31^. In the practical operation of controlling urban expansion, some measures have evolved, such as delineating the boundaries of urban growth, zoning, green belt policies and land use planning, to guide the rational growth of cities and control the disorderly spread^32–34^. As the problem of urban sprawl has intensified in China, the Chinese government has required local governments to draw red lines for the permanent protection of basic farmland and for ecological protection in order to solve the problems of losing cultivated land, deterioration of the ecological environment and urban sprawl^35,36^. Obviously, the premise of controlling the expansion of construction land is that we must understand the spatial distribution characteristics, trends and main drivers of construction land expansion. Then we combine the results of the study to formulate land management policies according to local conditions.

The research on the expansion of urban construction land in China has mainly focused on its characteristics and patterns, the influencing factors, the driving mechanisms and control strategies^37,38^. Scholars have carried out a considerable amount of research on the process of expansion, the spatiotemporal patterns, the characteristics of expansion and the evolutionary trends of construction land at the regional and urban levels^39,40^. Some scholars have also divided the expansion mode of urban construction into three types based on a landscape index: the marginal type, the filling type and the jumping type. Different types of urban construction land show different expansion patterns^41,42^. For example, Shanghai's early urban expansion was characterized by a single-core expansion mode; with the rapid development of the city, it gradually evolved into a "multi-core" expansion mode centered on satellite cities such as Pudong, Baoshan and Hongqiao^43^. In terms of the factors driving the expansion of urban construction land, economic factors are the most fundamental^44^. Some scholars have studied the development of macroeconomic conditions and urban expansion, and concluded that the rapid development of the urban economy and the improvement of residents’ income level are the fundamental driving forces promoting urban expansion^45,46^. Some scholars have also found that the expansion of China's urban construction land is closely related to the reform of the market economic system and the developmental trend of globalization^47^. In addition, social and policy factors have also had an important impact on promoting the expansion of urban land^48^. Natural factors also have an impact on the expansion of urban land^49^. The natural geographical environment is the most basic condition of urban development^50^. The regional, topographic, climatic and hydrological conditions of the city can directly affect the potential direction and mode of urban expansion^51^. For example, Chongqing City has been affected by the geographical environment, and its urban expansion presents a multi-center cluster style of development.

Scholars have carried out much research on the expansion of urban land in China. The main research methods are the urban expansion intensity index (UII), urban compactness and fractal dimensions^39,40^. The scope of research has mainly been limited to small-scale regions^43^. The content of the studies cover initial explorations of the process of how urban construction land expands and its impacts, revealing the driving mechanism of the expansion of urban land^10,20,21^. Then scholars paid more attention to the control of urban growth and evaluating the effects of the control measures, and then reflected on the scientific and rational process of polices for controlling urban growth^45–47^. As a rapidly urbanizing developing country, controlling the rapid expansion of China's construction land has also become an urgent problem. Most of the research results have a short research period^48^, and few studies have studied the spread of construction land in China in recent 30 years. In addition, there are few research results on the disorderly spread of urban construction land in China from the perspective of urbanization construction level. In light of this, this study used data on urban construction land in China from 1990 to 2020, combined with the spatial visualization function and statistical analysis methods of ArcGIS 10.2 and SPSS 19.0. This study examined and analyzed the evolutionary characteristics of the expansion of urban construction land in China and determined the expansion trends of urban construction land in China in the future. Combined with statistical analysis tools, the driving mechanism of the expansion of urban construction land was analyzed from the perspectives of the urban economic level, the population scale and the industrial structure in order to provide a scientific reference for controlling urban construction land, balanced allocation of the land use index and sustainable development in China and other developing countries.

The main structure of this study is as follows. Firstly, this study analyzed the status of expansion, the characteristics of spatial evolution, the speed of expansion and the future expansion trend of urban construction land in China from 1990 to 2020. We selected indexes related to urban construction and development to analyze the impact of urban construction and development on construction land in China. Finally, we put forward some suggestions for policies for controlling on urban construction land in China according to the characteristics of spatial evolution, the trends and the main factors influencing the expansion of construction land. Unlike most previous studies, this study creatively converts land-use raster data into comparable vector data. We combine spatial analysis with statistical analysis. Visually and dynamically expressing the spatial change characteristics and trends of construction land, it also excavates the impact of rapid urbanization on urban construction land spread. It fills in the blank of research on the impact of rapid urbanization on construction land expansion and provides scientific basis for the formulation of construction land control strategy. We propose five hypotheses from indicators related to urbanization, such as economy, population, industry and investment:

### Hypothesis H_1_

The urban economic level promotes the spread of construction land.

### Hypothesis H_2_

Urban population size has a positive correlation with construction land expansion.

### Hypothesis H_3_

Urban industrial scale can rapidly promote the spread of construction land.

### Hypothesis H_4_

There is a correlation between urban real estate development and the expansion of construction land.

### Hypothesis H_5_

City investment will also lead to the spread of urban construction land.

Based on these assumptions, we selected relevant urbanization construction indicators as shown in Table 1, and verified them through a multiple linear regression model to explore the impact of China's rapid urbanization on the spread of urban construction land. This study adds empirical research on the impact of urbanization on the driving force of urban sprawl and fills the gap in this field. In particular, this study covered 30 years of land use changes. This will help reveal the evolving characteristics of urban expansion and its driving factors in China.

**Table 1:** Selection and description of hypothetical influencing factors.

Hypothetical influencing factor	Index	Symbol	Unit	Average	Standard deviation
	Urban construction land area	Y	km^2^	1238.17	2294.64
Urban economic level	Per capita GDP of the city	X_1_	Yuan	63,090.62	36,115.96
Urban population	Registered population of residents at the end of the year	X_2_	10^4^ people	447.66	331.39
Urban industry	The proportion of employees in the secondary industry in the city	X_3_	%	62.41	422.08
Urban construction industry	Number of persons employed in urban units in the construction industry in the whole city at the end of the year	X_4_		80,557.45	131,083.81
Urban investment	Investment enterprises from Hong Kong, Macao and Taiwan throughout the city	X_5_		66.37	210.8
	Foreign investment enterprises throughout the city	X_6_		79.62	257.48

## Methods and materials

### Research methods

#### Urban expansion intensity index (UEI)

The UEI calculates the average annual growth rate of construction land between two time points to characterize the speed of development and the strength of the city^52^. The formula is

1 $$UEI=\frac{{S}_{m+n}-{S}_{m}}{A}\times \frac{1}{n}\times 100\%$$

In this formula, *UEI* is the index of the city's expansion intensity. ${S}_{m+n}$ and ${S}_{m}$ are the construction land area at the two time points. *A* is the total area of urban land. *n* is the length of the study period. The smaller the *UEI*, the weaker the intensity of urban expansion and the slower the urban development. The intensity of urban expansion is divided into five grades according to relevant studies^53^: 0 < *UEI* ≤ 0.28, slow expansion; 0.28 < *UEI* ≤ 0.59, low-speed expansion; 0.59 < *UEI* ≤ 1.05, medium-speed expansion; 1.05 < *UEI* ≤ 1.92, rapid expansion; *UEI* > 1.92, high-speed expansion.

#### Analysis of hotspots

The purpose of this study was to describe the evolutionary patterns of the expansion of urban construction land in China from 1990 to 2020 in detail. Based on the data on the expansion of urban construction land, the Getis–Ord Gi* method was used to judge the relationships of the expansion of the urban construction land in different locations^54^. The results are visualized as hotspots, insignificant areas and cold spots to determine the stages of expansion.

2 $${{G}_{i}}^{*}\left(q\right)=\frac{{\sum }_{n=0}^{m}{w}_{in}\left(q\right){x}_{n}}{{\sum }_{n=0}^{m}{x}_{n}} .$$

In order to facilitate a comparative analysis, we standardized ${{G}_{i}}^{*}\left(q\right)$, so the formula above became

3 $$R\left({{G}_{i}}^{*}\left(q\right)\right)=\frac{{{G}_{i}}^{*}\left(q\right)-E\left({{G}_{i}}^{*}\left(q\right)\right)}{\sqrt{Var\left({{G}_{i}}^{*}\left(q\right)\right)}},$$

where $E\left({{G}_{i}}^{*}\left(q\right)\right)$ denotes the mathematical expectation of ${{G}_{i}}^{*}\left(q\right)$, and $Var\left({{G}_{i}}^{*}\left(q\right)\right)$ represents the variance of ${{G}_{i}}^{*}\left(q\right)$. If $R\left({{G}_{i}}^{*}\left(q\right)\right)$ is positive and statistically significant, this means that the expansion of urban construction land *i* is located in the a hotspot area of agglomeration. If $R\left({{G}_{i}}^{*}\left(q\right)\right)$ is negative and the statistical result is significant, this means that the expansion of urban construction land *i* is located in a cold spot area of agglomeration.

#### Nuclear density

Kernel density is often used to measure the density of an object within a grid cell. This study calculated the density distribution of the expansion of urban construction land area according to the data of each city’s construction land area and generated a continuous core density value of the surface area^55^. We explored the characteristics of the spatial distribution of the expansion of construction land in China from a quantitative perspective. The higher the value of nuclear density, the greater the concentration of areas with the expansion of construction land, and vice versa. The formula for calculating nuclear density is

4 $${\widehat{\lambda }}_{r}\left(p\right)=\sum_{n=1}^{m}\frac{3}{\uppi {r}^{4}}{\left[1-\frac{{\left(p-{p}_{n}\right)}^{2}}{{m}^{2}}\right]}^{2},$$

where* p* represents the city whose density needs to be calculated, ${\widehat{\lambda }}_{r}\left(p\right)$ represents the estimated nuclear density of the city and *p*_*n*_ is the position of the *n*th city in the circular region with *r* as radius and *p* as center, that is, the width of the extension of the surface area in space starting from *p*.

#### Multiple linear regression model

In order to deeply explore the potential influencing factors of the spatial differences in the distribution of the expansion of urban construction land, we used the multiple linear regression analysis and correlation analysis methods in SPSS19.0 software^56^. We constructed an explanatory model of the spatial distribution of construction land in China and the related influencing factors such as the urban economic level, the size of the population, the industrial structure and foreign investment (Table 1). The robustness and the degree of fit of the model were tested, and the explanatory variables were used to explain the evolution mechanism of the spatial differences in the distribution of construction land in China. We hope that the interpretative model will provide a reference for the Chinese government to control the disorderly spread of urban construction land in China. Firstly, we used SPSS19.0’s correlation analysis tool to analyze the factors influencing the differences in the distribution of construction land. The significantly correlated variables affecting the expansion of construction land were selected. Secondly, we performed multiple linear regression analyses of significantly correlated variables using forced entry models. We gradually eliminated the urban residential land area, urban administrative areas, investment in the construction of urban infrastructure and other variables with poor collinearity and weak correlations. Finally, the interpretative model with a high degree of fit, strong collinearity and significant correlations were obtained. The expression of the interpretative model is

4 $${Y}_{n}={\alpha }_{n}+\sum_{i=1}^{m}{\beta }_{ni}{X}_{ni}$$

In this formula, ${Y}_{n}$ represents the construction land area of city *n, β* is the coefficient of each explanatory variable, *α* is a constant and *X* is the value of the explanatory variable.

### Data collection

The research data included Chinese land use data and data from the statistical yearbook on urban construction. Among them, the maps are based on the standard map produced under the supervision of the Ministry of Natural Resources of the People's Republic of China: Approval number GS (2020) 3183 (http://bzdt.ch.mnr.gov.cn/). The land use data were obtained from the Resources and Environmental Science and Data Center of the Chinese Academy of Sciences (https://www.resdc.cn/), accessed on 1 December 2023. The land use data are the national land use raster data with a spatial resolution of 1 km × 1 km. The land use cover change (LUCC) classification system was used for the national land use raster data, and the three-level classification system was adopted^57^. Firstly, we merged the raster data. Secondly, the information on the land use classification was integrated. The land was classified into six classes: forest land, cultivated land, grassland, construction land, water bodies, unused land and sea^58^. Finally, we repeated the first two steps to obtain the urban construction land data for 1990, 1995, 2000, 2005, 2010, 2015 and 2020. Another type of data came from China Urban Construction Statistical Yearbook (2020). The data mainly included statistics on China's urban economic income, population, industry and employment (Table 1)^59^. What needs to be explained here is that the statistical data for Taiwan, Hong Kong and Macao are updated slowly. This resulted in missing statistics for these regions. Therefore, we did not carry out statistical research on Taiwan, Hong Kong and Macao.

## Characteristics of the expansion of construction land in China

### Characteristics of overall change and utilization

**Figure 1:**
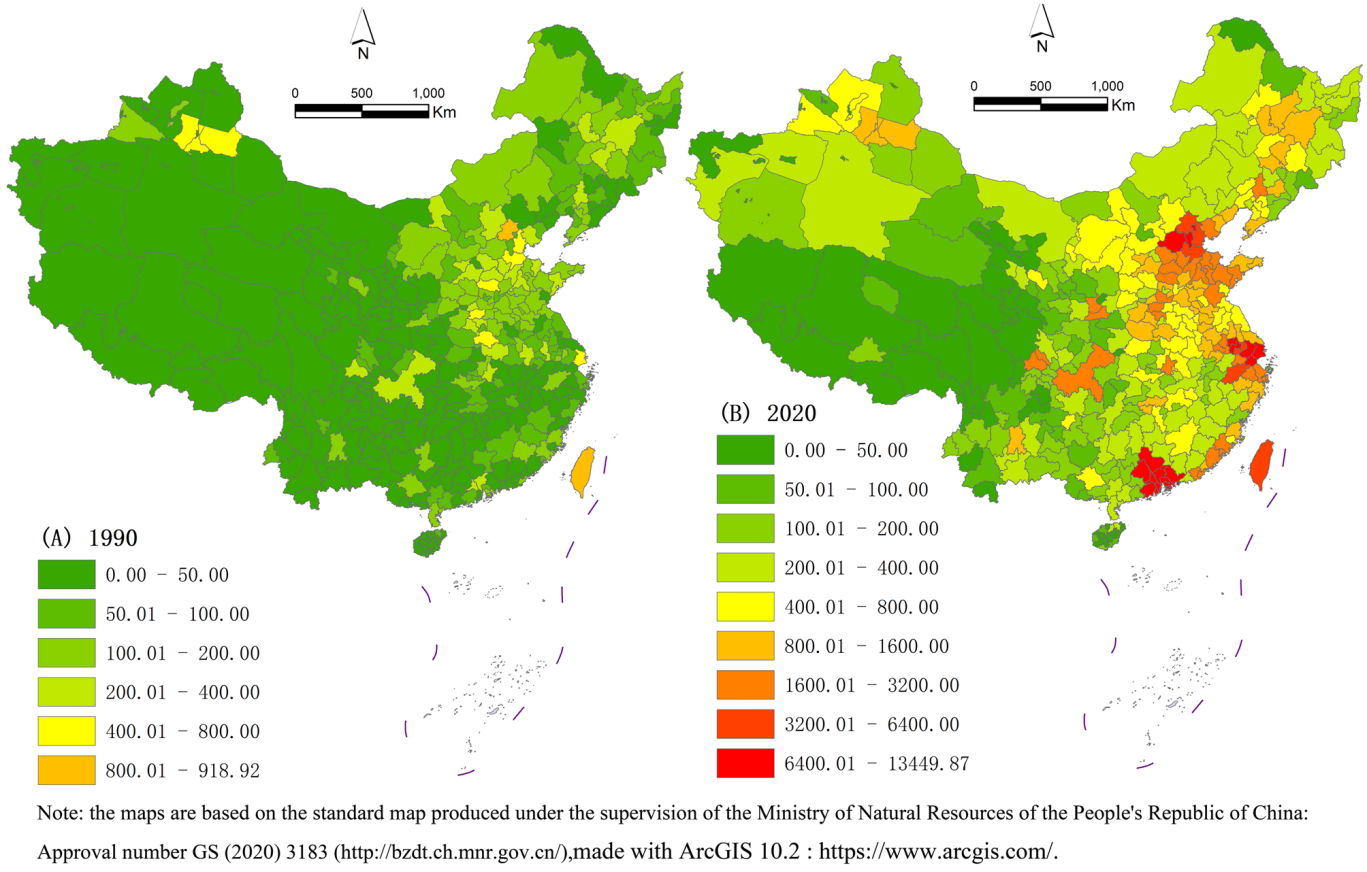
Distribution of total area of urban construction land in (A) 1990 and (B) 2020.

From 1990 to 2020, the total amount of urban construction land in China increased by about 13 times (Fig. 1, Table 2). Among the regions, the total growth of urban construction land in Suzhou City ranked first in China. Those ranking second were Qingyuan, Huizhou, Zhaoqing, Jiangmen, Zhuhai, Shenzhen, Zhongshan, Dongguan, Foshan, Guangzhou and Shanghai, in turn. The total area of construction land in these cities has increased by more than 10000 km^2^. In terms of total growth, the growth of urban construction land in China is mainly concentrated in the Yangtze River Delta, the Pearl River Delta and the Beijing–Tianjin–Hebei urban agglomeration^60^. The expansion of construction land in the Pearl River Delta urban agglomeration is the most obvious. From the per capita perspective, Zhuhai City has the most construction land area per capita. Qingyuan City, Zhaoqing City, Zhongshan City and Zhenjiang City ranked in the top five. The Pearl River Delta's overall construction land area per capita also ranked first in the country. From the perspective of GDP output efficiency, Qingyuan City in Guangdong Province needed to consume 6.19 km^2^ of construction land for every CNY100 million GDP output (Table 2). Its construction land’s efficiency output GDP was the lowest, which also reflects that the efficiency of urban construction land use in Qingyuan City is relatively low, and there is still much room for improvement in terms of land use efficiency^61^. Zhaoqing City, Jiangmen City, Zhongshan City, Zhuhai City, Huizhou City and Baoding City in Hebei Province; Langfang City and Siping City in Jilin Province and Zhenjiang City of Jiangsu Province ranked next. The utilization efficiency of construction land in these cities was also at a relatively low level for the whole country. The waste of urban construction land resources is serious, and the economic benefit of the output is low. There is large room for improvement. Obviously, China has experienced rapid development in the past few decades. Only Shenzhen, Shanghai and Guangzhou and other first-tier cities have experienced reasonable expansion of their construction land. Shanghai, North Shenzhen and Guangzhou are the first-tier cities with the highest intensity of construction land use. The intensity of construction land use in Shandong Peninsula and the eastern coastal areas was generally high. The intensity of Xinjiang’s and Xizang’s construction land use was relatively low. These first-tier cities showed efficient and intensive land use patterns, both in terms of per capita land area and GDP output efficiency. However, most Chinese cities present the problem of wasting land resources, mainly manifested as the disorderly spread, the low land utilization rate and low output efficiency.

**Table 2:** Statistics of the changes in construction land use in China.

Ranking	City	Growth in construction land area from 1990 to 2020, km^2^	City	Per capita construction land area in 2020	City	Land area consumed by the generation of CNY100 million GDP
Top ten	Suzhou	13,356.3	Zhuhai	43.97	Qingyuan	6.19
	Qingyuan	10,953.71	Qingyuan	27.66	Zhaoqing	4.75
	Huizhou	10,953.08	Zhaoqing	26.70	Jiangmen	3.40
	Zhaoqing	10,944.98	Zhongshan	24.02	Zhongshan	3.36
	Jiangmen	10,863.87	Zhenjiang	23.48	Zhuhai	3.09
	Zhuhai	10,698.16	Jiangmen	22.79	Huizhou	2.61
	Shenzhen	10,626.77	Huizhou	18.19	Baoding	2.10
	Zhongshan	10,565.47	Jiaxing	13.14	Langfang	2.02
	Dongguan	10,554.96	Changzhou	12.35	Siping	2.02
	Foshan	10,452.43	Langfang	12.27	Zhenjiang	1.78

### Analysis of the characteristics of spatial evolution

In the past 30 years, the upsurge in land marketization in China has rapidly promoted the spread of urban construction land^62^. This study aimed to characterize the spatial evolution of urban construction land in China from 1990 to 2020. The data on urban construction land in 1990, 1995, 2000 and 2005 were selected, and the kernel density analysis method of ArcGIS 10.2 was used. We selected suitable step intervals to analyze the characteristics of the spatial evolution of urban construction land in China. The search radius *r* was 0.5 km, and the area of land sold was used as the weight. We used a custom method to divide the nuclear density values to compare and analyze the nuclear density of construction land between years.

**Figure 2:**
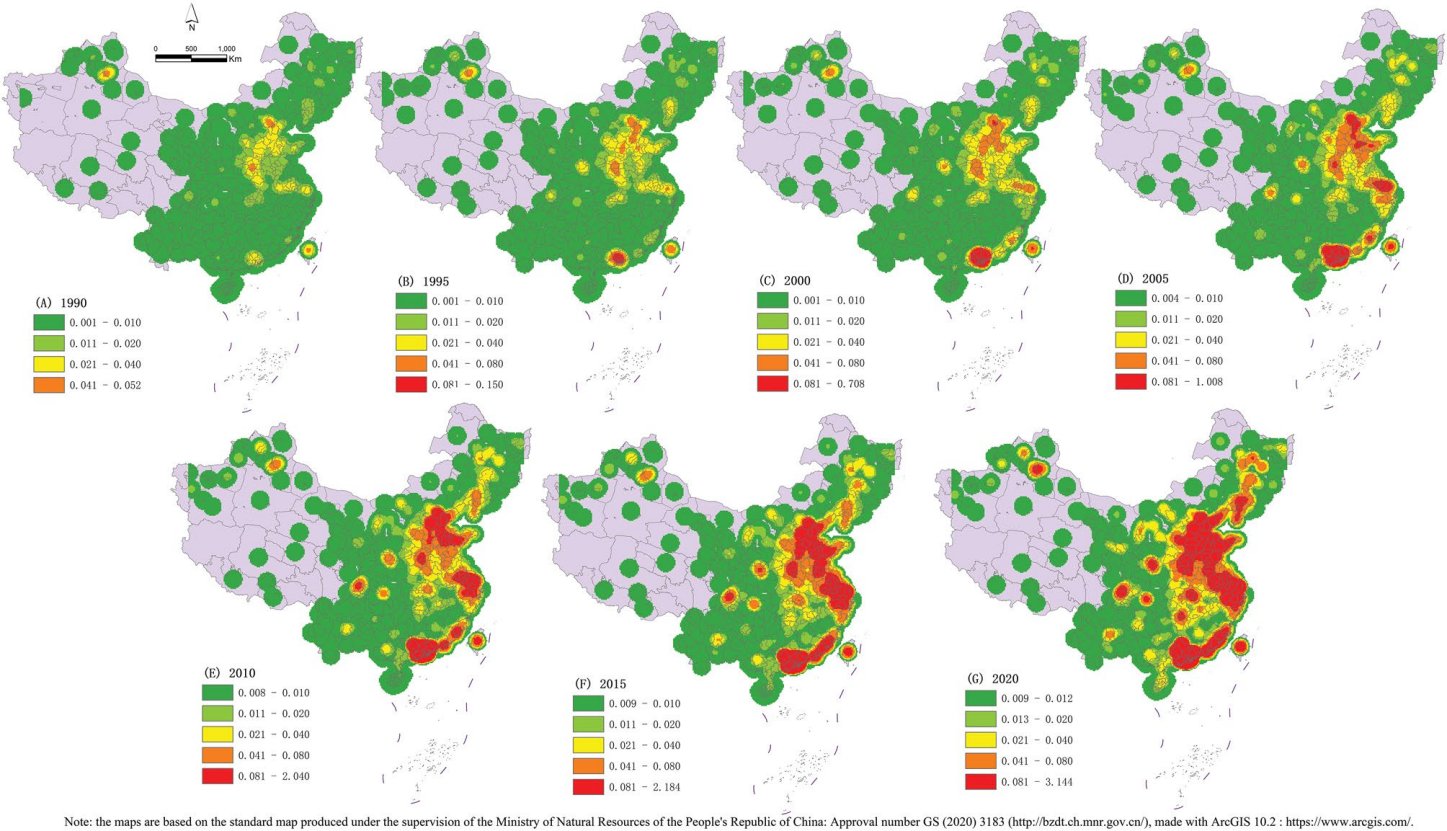
Distribution map of nuclear density of urban construction land in (**G**) 2020, (**F**) 2015, (**E**) 2010, (**D**) 2005, (**C**) 2000, (**B**) 1995 and (**A**) 1990.

The results showed that the urban construction land in China has a "point–line–area" expansion pattern, forming spatial evolution stages of point agglomeration, a linear series, and spread of the area (Fig. 2). It showed a hierarchical and multi-core circle expansion structure. The expansion process of urban construction land showed a circular pattern of highly concentrated core cities, dense surrounding cities and sparse districts and counties. The center of gravity of nuclear density also showed the characteristics of spatial evolution from west to east, and from north to south. In 1990, the construction land gradually formed a point agglomeration pattern, with Beijing, Shenzhen and Shanghai as the core. With the passage of time, other provincial capitals gradually became cores and drove the expansion of construction land in the surrounding cities. In 2005, the density of the core urban construction land reached its peak value. The benefits of land expansion began to drive the growth of the surrounding provinces and cities, and a linear series pattern gradually formed. In 2005, three belts of expanding construction land gradually formed, namely Shenzhen–Xiamen–Fuzhou, Beijing–Shijiazhuang–Zhengzhou-" and Beijing–Jinan–Shanghai. As the scale of the surrounding urban construction land continued to grow, the expansion trend of construction land spread rapidly to major cities in China, gradually forming a planar spreading pattern. In 2020, China's construction land had gradually formed a planar spreading pattern, headed by the Pearl River Delta, Yangtze River Delta, Beijing–Tianjin–Hebei and Central Plains urban agglomerations. From the perspective of the "point–line–area" pattern of expansion, the expansion rate of urban construction land in eastern China was significantly higher than that in western China. The urban construction land in western China has not formed an obvious pattern of zonal series and is still in the core agglomeration stage. Obviously, there is an obvious structural imbalance in the expansion of urban construction land between the east and west of China.

### Speed and trend of expansion

**Figure 3:**
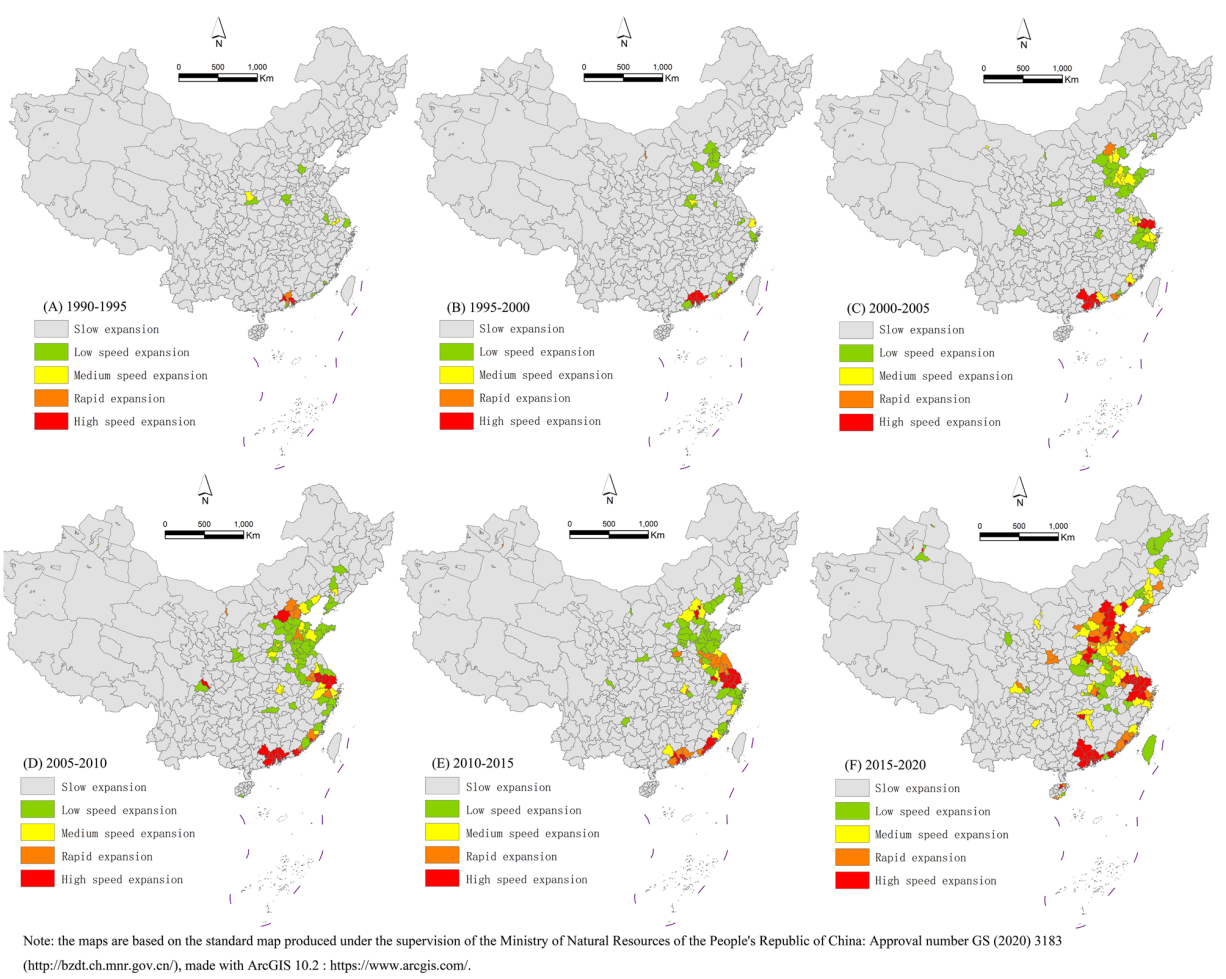
Speed of land expansion in (**F**) 2015–2020, (**E**) 2010–2015, (**D**) 2005–2010, (**C**) 2000–2005, (**B**) 1995–2000 and (**A**) 1990–1995. ArcGIS 10.2: https://www.arcgis.com/.

This study aimed to further characterize the speed of expansion of urban construction land in China. We used the UEI to measure the urban construction land in stages and calculated the annual average growth rate of urban construction land at two time points (Fig. 3)^52,53^. The results showed that the cities with high-speed expansion in China were mainly concentrated in the Pearl River Delta region from 1990 to 2000. After 2000, a medium-speed expansion belt along the southeast coast gradually formed, and after 2015, China's urban expansion belt gradually moved inland. From the perspective of the speed of expansion, it also showed a circular expansion mode of a high-speed core, a medium-speed periphery and a low-speed periphery. From 1990 to 1995, Macao, Dongguan, Hong Kong, Shenzhen and Foshan were in the stage of high-speed expansion. Zhongshan City and Guangzhou City were in the stage of rapid expansion. The expansion speed of construction land in other cities was relatively slow. Between 2015 and 2020, there were 42 cities expanding at high speed and 31 cities expanding rapidly. The number of cities with high-speed expansion and rapid expansion has increased significantly. Among them, Macao, Hong Kong, Shenzhen, Zhongshan, Dongguan, Foshan and other cities have maintained a stage of sustained high-speed expansion over 30 years. Jiaxing, Suzhou, Qingyuan and Xiamen have also begun to expand greatly over the past 10 years. The high-speed expansion of cities still concentrated in the Yangtze River Delta, Pearl River Delta and Beijing–Tianjin–Hebei urban agglomerations, which also shows the spillover phenomenon of the expansion of urban construction land. The rapidly expanding cities gradually showed the trend of transferring to the Central Plains, the Yangtze River Central Plains and the Chengdu–Chongqing urban agglomeration.

**Figure 4:**
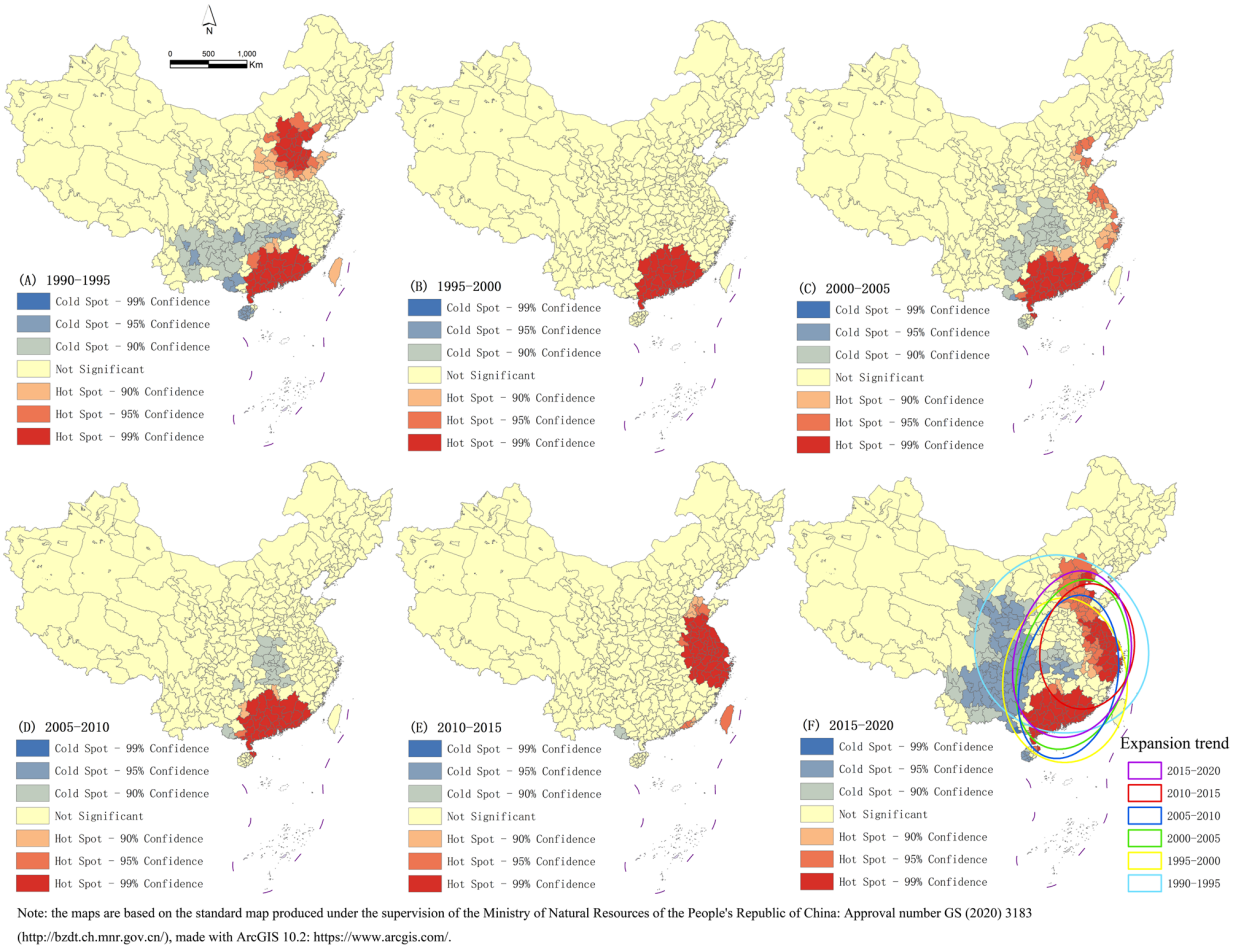
Trends of the cold spots and hotspots of expansion in (**F**) 2015–2020, (**E**) 2010–2015, (**D**) 2005–2010, (**C**) 2000–2005, (**B**) 1995–2000, (**A**) 1990–1995. ArcGIS 10.2: https://www.arcgis.com/.

The distribution of cold spot and hotspots of the expansion of urban construction land (Fig. 4) shows that at the beginning of China's reform and opening up, the hotspots of the expansion of construction land were mainly concentrated in the Pearl River Delta and Beijing–Tianjin–Hebei regions. With the success of the opening up of cities such as Shenzhen and Guangzhou, the hotspots of the expansion of urban construction land moved south after 1995. Since China's accession to the WTO in 2001, China has begun to participate extensively in economic globalization and has increased its opening to the outside world and regions^63^. China's southeast coastal cities have been opening up to carry out transnational trade activities. As a result, the construction land of southeastern coastal cities has expanded rapidly, typical representatives of which are Shanghai, Xiamen and Qingdao. In September 2010, President Xi Jinping proposed to jointly build the Silk Road Economic Belt and the 21st Century Maritime Silk Road, known as the Belt and Road Initiative, both aimed at strengthening coordinated regional development. The Yangtze River Delta is rising rapidly as the bridgehead of the Belt and Road Initiative. This has also led to the rapid expansion of urban construction land in the Yangtze River Delta region, which has become a new hotspot area for the expansion of construction land. At this time, the center of gravity of construction land expansion shifted eastward. After 2016, the national investment in foreign trade slowed down and the economic pressure was great. In order to reduce costs and increase efficiency, the Pearl River Delta, Beijing–Tianjin–Hebei and Yangtze River Delta regions began to implement the industrial restructuring strategy of retreating from secondary industries to tertiary industries. The first-tier cities began to relocate secondary industry to the underdeveloped cities around them, and vigorously developed tertiary industry themselves. A large number of industrial relocations has led to the rapid expansion of construction land in inland cities. At this stage, the hotspot area of the expansion of construction land moved inland. To sum up, the improvement in the economic level and adjustment of the industrial structure of coastal cities also promoted the rapid expansion of construction land in inland cities. Construction land sprawl in inland cities of China will become a trend in the future.

## Analysis of the influencing factors

We finally get the main variables with a high degree of fit, strong collinearity and significant correlations were obtained through multiple regression analysis (Table 3).

**Table 3:** Multiple linear regression analysis of urban construction land area and the related influencing variables in China.

Model	Hypothetical influencing factor	Variable	Sig	T	B	VIF
Dependent variable: urban construction land area		(constant)	0.005	− 2.832	− 683.047	
	Urban economy	Per capita GDP of the city, CNY (*X*_1_)	0.000	5.052	0.016	1.897
	Urban population	Registered residence population at the end of the year/10^4^ people (*X*_2_)	0.000	4.685	1.557	1.726
	Urban industry	The proportion of employees in the secondary industry in the city/% (*X*_3_)	0.000	4.562	0.917	1.024
	Urban construction industry	Number of persons employed in the construction industry in urban units at the end of the year in the whole city (*X*_4_)	0.000	− 4.201	− 0.004	2.021
	Urban investment	Investment enterprises from Hong Kong, Macao and Taiwan throughout the city/ (*X*_5_)	0.000	8.240	4.754	2.104
		Foreign investment enterprises throughout the city (*X*_6_)	0.000	3.540	1.762	2.336

**Figure 5:**
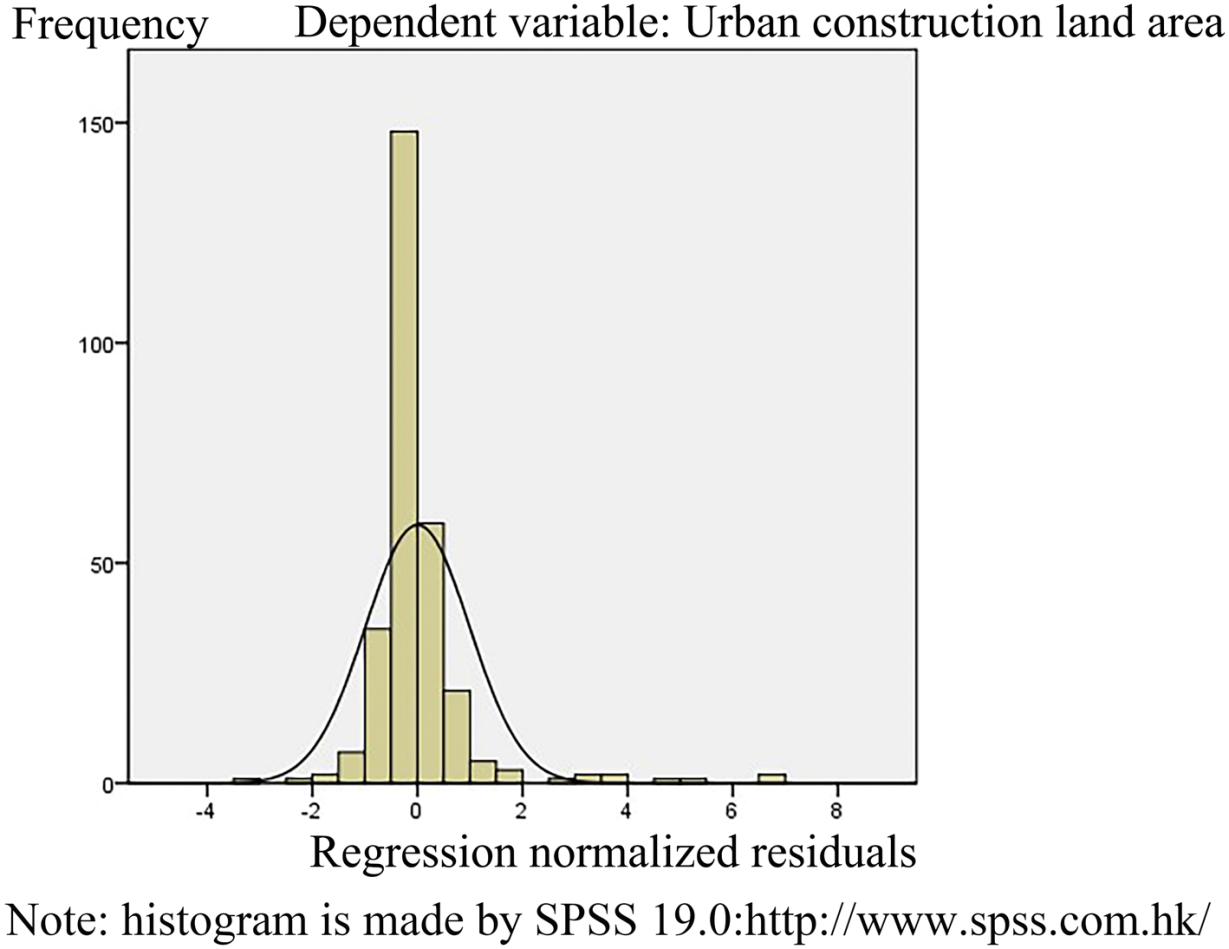
Standardized residual histogram.

It is not difficult to see from Table 3 that the absolute T-test values of the explanatory model were all greater than 1.96, indicating that explanatory variables had an obvious influence on multiple linear regression models^56^. The Sig. values of the explanatory variables were also less than 0.05, which proves that there was a significant correlation between the explanatory variables and the dependent variables. In addition, the collinearity (VIF) values of the explanatory variables were all less than 7.5, which indicated that there was no collinearity among the explanatory variables. We also found that the Sig. value of the explanatory model was 0.000^a^ by a variance test, which indicated that heteroscedasticity issues did not exist and also showed that the explanatory model had strong statistical significance. Furthermore, the model passed the robustness test, and the R^2^ value of the interpretation model was not less than 0.5, which further proved that the interpretative model has high robustness, a high degree of fit and good quality. The residual histogram of the interpretative model also exhibited a normal distribution pattern (Fig. 5), further illustrating the statistical significance of the interpretative model. It can be used to explain the main driving forces of the differences in the expansion of urban construction land in China. The explanatory model is

5 $$Y = - {683}.0{47 } + \, 0.0{16}X_{{1}} + { 1}.{557}X_{{2}} + 0.{917}X_{{3}} - \, 0.00{4}X_{{4}} + { 4}.{754}X_{{5}} + { 1}.{762}X_{{6}} ,$$

It is not difficult to see from the interpretation model that the urban economy, the urban population, urban industry, the construction industry and foreign investment have significant correlations with the expansion of urban construction land. Among these, the scale of foreign investment enterprises in cities had the most significant impact on the expansion of construction land. When other variables remained unchanged, for every additional investment enterprise from Hong Kong, Macao and Taiwan, the city increased by 4.754 km^2^ of construction land. Proper control of the number of foreign investment enterprises will help control the disorderly spread of cities. In particular, cities with rapidly expanding economies should pay more attention to controlling the growth rate of foreign-funded enterprises. Reducing the dependence of cities on foreign investment helps slow down the expansion of urban construction land to control urban sprawl. The urban population also has a significant impact on the expansion of urban construction land. For every 10,000 registered people in the population, the urban construction land area will increase by 1.557 km^2^. Obviously, the growth of the registered urban population drives the settlement of houses and demand for the expansion of construction land. Therefore, proper control of the population would help to alleviate the pressure of shortages of urban construction land. Secondly, the proportion of employees in the urban secondary industries also had a positive impact on the expansion of urban construction land. The greater the proportion of employees in the urban secondary industries, the greater the demand for urban construction land. Local governments can properly control the scale of secondary industries and eliminate secondary industries with low production efficiency and overcapacity, which would be conducive to intensive, economical and efficient use of urban land and the green sustainable development of cities. GDP per capita also had a significant impact on the expansion of urban construction land. For every CNY 1 increase in GDP per capita, the area of urban construction land will increase by 0.016 km^2^. As per capita income increases, people's demand for high-quality living space will increase, which will require more construction land to meet these high living standards. Obviously, the urban economic level also has a positive impact on the expansion of urban construction land. In addition, employment in the urban construction industry and the expansion of urban construction land were negatively correlated. From the explanatory variable’s coefficient, we can see that with more urban construction practitioners, the expansion of urban construction land is slower. This can only show that the city is still in the early stage of spread, and the scale of the urban construction industry needs to be controlled in time to prevent the rapid spread of the city in the later stages.

In brief, the urban economy, population, industry, construction and foreign investment are the key factors in the spatial differences in the distribution of how urban construction land is expanding in China. The relationships between these factors and the changes in urban construction land area are positive and negative. Changes in any explanatory variable in the model will affect the speed and scale of urban sprawl. According to the explanatory variables of the model, each city can formulate an expansion strategy for urban construction land that is suitable for its own conditions. Only local governments can achieve the goal of controlling urban sprawl according to the local conditions and the city’s policies.

## Discussion

### Strictly control the expansion speed of urban construction land and formulate output efficiency strategy

Most Chinese cities present the problem of wasting land resources, mainly manifested in disorderly sprawl, low land utilization rate, and low output efficiency^64^. Local governments should formulate evaluation indicators for construction land utilization efficiency and output efficiency according to local conditions^65^. For example: Qingyuan, Huizhou, Zhaoqing, Jiangmen, Zhuhai, Zhongshan, Dongguan, Zhenjiang, Jiaxing, Baoding and Langfang and other second-tier cities have large land use scale, large per capita areas and low economic output benefits (Table 2). The central government should formulate the construction land supply index by stages according to the urban development stage^66^. Properly control the supply area of these urban construction land, force local governments to redevelop inefficient land, and improve the utilization efficiency of existing land^67^. In addition, the local government needs to formulate detailed land output benefit objectives, and the central government supervises and evaluates the development process of local construction land according to the output benefit objectives^68^. The government shall impose fines, rectification, demolition and reconstruction on the development and construction activities that have not been completed^69^. Fund subsidies and land use index inclination will be given to projects that overfill land output benefits^70^. Only through the land management measures with clear rewards and punishments can we improve the efficiency of land use and control urban sprawl. These cities should rely on direct government intervention and indirect market regulation, and should be dominated by government intervention such as taxation and regulations. Suzhou, Foshan, Hangzhou and other new first-tier cities have large land use scale and high economic efficiency. But they are too big per capita. This also shows that the land use efficiency of these new first-tier cities is still large^71^. Local governments should pay attention to increasing population size and properly improving the artificial use efficiency of land in the process of development and construction. Shenzhen, Guangzhou, Shanghai and Beijing and other first-tier cities have large land use scale, high utilization efficiency and small per-capita areas. Although these first-tier cities have high land use efficiency, they should also pay attention to ecological environmental problems. A large number of construction land spreads also destroyed a large number of vegetation and cultivated land, seriously threatening the safety of the regional ecological environment^72^. Local governments should continue to pursue land use efficiency rather than blindly expanding the scale of construction land. Therefore, local governments in first-tier cities need to strictly control land development indicators, control the disorderly spread of urban construction land, and protect vegetation and cultivated land area, to maintain regional ecological environment balance and realize regional sustainable development.

### Optimize the allocation of regional resources and reasonably delimit the boundary of urban growth

In the process of the expansion of urban construction land, urban construction land as a whole presents a pattern of "point-line-area" expansion. Planar expansion is mainly concentrated in the Yangtze River Delta, Beijing-Tianjin-Hebei, Pearl River Delta and Central Plains urban agglomeration. Among them, the compactness of urban expansion of Central Plains Urban Agglomeration is on the low side as a whole^73^. Hebei Baoding, Hengshui, Linyi, Xingtai and other cities in the Central Plains need to strictly control the urban growth boundary. Their urban boundaries show a clear phenomenon of sprawl. Sprawl is more likely to destroy land integrity than inefficient output^74^. Local governments should strictly delimit the boundaries of urban growth to control the ecological damage caused by urban sprawl^75^. In addition, Guizhou Anshun City, Shaanxi Tongchuan City, Sichuan Bijie City and other western cities also showed the phenomenon of disorderly spread. Most of these cities are concentrated in the underdeveloped areas of southwest and northeast China. In addition to some of these cities being restricted by mountainous conditions, most of them have led to the disorderly expansion of urban construction land boundaries due to their low economic level and insufficient control consciousness. These city governments should also make rational use of construction land management and control tools such as urban construction land growth boundaries, total construction land control, green belt around the city, and basic farmland protection system^76^. To reduce the occupation of basic farmland caused by the disorderly expansion of urban construction land, reduce the disorderly expansion of cities, and promote the transformation of cities from high investment to high-quality development.

### Adjust urban construction development indicators and control the rational distribution of urban construction land

We find that GDP per capita, urban population size, urban secondary industry employee size, construction industry size and foreign investment scale have significant correlations with the expansion of urban construction land. Local governments can control the expansion of urban construction land by appropriately controlling the urban population scale, foreign investment scale and construction scale in combination with the current urban development stage^77^. There is a significant gradient in the expansion speed of urban construction land in China, and the coastal area is higher than other areas^78^. Governments in coastal areas may appropriately control such indicators as the scale of foreign investment and population size to encourage foreign investment to shift to central and western regions^79^. With the rise of cities in the central and western regions, China's economic center of gravity will move inland, and population and industry will also shift to the central and western regions. In this process, coastal cities not only achieve the goal of rapid urban sprawl, but also have the opportunity to redevelop inefficient land in the region and improve land use efficiency^80^. Of course, in the process of undertaking foreign investment and industrial transfer, the central and western regions need to strictly and carefully assess the efficiency of land use and output benefits^81^. Local governments should refuse inefficient industries to occupy a large amount of construction land^82^. Especially Qingyuan, Zhaoqing, Jiangmen, Zhongshan, Zhuhai, Huizhou, Baoding, Langfang, Siping and Zhenjiang should actively improve the efficiency of urban construction land and increase output, move closer to the surrounding first-tier cities, introduce high-efficiency and high-output industries, and eliminate the abuse of urban construction land at the source of investment attraction. In the areas with a poor endowment of natural resources in the central and western regions, the value of urban construction land should be fully developed, and inefficient or even ineffective new urban construction land should be reduced as much as possible^83^. Local governments should re-plan the urban development of the old industrial areas in Northeast China and promote the transformation from secondary industries to tertiary industries^84^. The government should take the lead in renovating inefficient urban land and transforming various types of land used for old industries into efficient urban construction land in combination with relevant projects^85^.

### Encourage diversified development and mixed-use of land

With the advancement of the new urbanization process in China, urban land use needs to be diversified gradually^86^. Cities should allocate all kinds of land resources effectively, emphasizing the mixed use of land and intensive development strategy^87^. Local governments focus on compact planning of urban construction land, forming a smart growth model of urban development. The construction of new urban areas needs to actively promote the integrated development of industries and cities, increase infrastructure and public service facilities, realize balanced coordination of land use functions and overcome urban diseases^88^. Old city reconstruction needs to increase the green space, square, and other public spaces, realize the overall balance of land use function, and meet the needs of residents. Our diversified use of construction land can effectively solve urban problems such as urban congestion and deterioration of the ecological environment.

### Policy adjustment according to local conditions to achieve smart growth

The analysis of the expansion characteristics of urban construction land in China shows that there are great differences in the area, structure and growth rate of urban construction land in different regions, provinces and grades of China^89^. We suggest that differentiated urban expansion governance and land use control measures be tailored to different regions, provinces and cities of different sizes and grades^90^. From the perspective of total amount control of urban expansion, the Pearl River Delta, Yangtze River Delta, Beijing-Tianjin-Hebei and Central Plains urban agglomeration should be the key prevention areas. From the perspective of urban expansion speed, the growth focus of urban construction land has gradually shifted from eastern provinces to central and western provinces. Therefore, the focus of urban expansion growth control should be on the central and western provinces. At the same time, the city scale grade is also one of the issues that needs to be considered in urban expansion governance. China's first-tier cities and new first-tier cities consume the most urban construction land, so more urban construction land control targets should be targeted at these cities. For these cities, we should formulate the strictest measures to control the total amount, force them to revitalize their stocks, update their current quantities, and promote the organic renewal and smart growth of cities^91^.

## Conclusion

Based on the data of urban construction land in China from 1990 to 2020, this study analyzed the process of the expansion of urban construction land in China from the perspectives of the characteristics, speed and trend of expansion and the influencing factors. The following conclusions were made:

1. China's construction land shows the characteristics of disorderly spread, a low land utilization rate and a low output efficiency, and it shows the remarkable characteristics of being high in the east and low in the west, being mostly concentrated in the Pearl River Delta, Yangtze River Delta and Beijing–Tianjin–Hebei urban agglomerations. Shanghai, Beijing, Shenzhen, Guangzhou and other first-tier cities have the highest intensity of construction land use. The intensity of construction land use in Shandong Peninsula and the eastern coastal areas is generally high. The intensity of Xinjiang’s and Xizang’s construction land use is relatively low. Generally speaking, the expansion of and change in construction land in China should not only keep pace with the requirements of developing construction land in the Five-Year Plan but should also echo China's main economic development policies and developmental processes. These findings indicate that land policies at different development stages have significant effects on the expansion of construction land.
2. The expansion of China's construction land also presents a "point–line–plane" evolutionary pattern, with the changing stages of point-like aggregation, a linear series and planar spread. In the early stage, Shenzhen, Shanghai, Guangzhou and Beijing were the core cities for agglomeration and expansion, gradually forming a zonal pattern of spread along the southeast coast. After 2015, a planar pattern surrounding the Pearl River Delta, Yangtze River Delta, Beijing–Tianjin–Hebei and Central Plains urban agglomerations formed.
3. The expansion of urban construction land is significantly correlated with the urban economic level, population size, industrial structure, foreign investment and land policy. The overall spatiotemporal evolution is the final result of the interactions among all these factors. The overall distribution pattern of the expansion of urban construction land is basically consistent with the distribution of the major urban development clusters and the current economic development trends of various regions in China. According to these key factors, local governments should formulate strategies to control the disorderly spread of urban construction land according to the local conditions, and achieve the sustainable development goal of land conservation and efficient use.

This study analyzes the characteristics, expansion trend and influencing factors of construction land spread in China, but there are some shortcomings in the study. the multiple linear regression model is limited to capturing the temporal dynamics of expansion driving forces. We will next consider the panel data model and analyze the drivers of construction land disorder. In addition, the driving forces of urban expansion varies among cities of different sizes, eastern coastal cities and inland cities. We will further explore the differential driving mechanism of urban expansion in different regions and different types of cities, and continue to refine the research content. This is also the direction that this research needs to go further.

## Data Availability

All data included in this study are available upon request by contact with the corresponding author.
